# Selective Autophagy of the Protein Homeostasis Machinery: Ribophagy, Proteaphagy and ER-Phagy

**DOI:** 10.3389/fcell.2019.00373

**Published:** 2020-01-21

**Authors:** Carsten J. Beese, Sólveig H. Brynjólfsdóttir, Lisa B. Frankel

**Affiliations:** ^1^Biotech Research and Innovation Centre, University of Copenhagen, Copenhagen, Denmark; ^2^Danish Cancer Society Research Center, Copenhagen, Denmark

**Keywords:** ribophagy, proteaphagy, ER-phagy, selective autophagy, protein homeostasis, ubiquitin

## Abstract

The eukaryotic cell has developed intricate machineries that monitor and maintain proteome homeostasis in order to ensure cellular functionality. This involves the carefully coordinated balance between protein synthesis and degradation pathways, which are dynamically regulated in order to meet the constantly changing demands of the cell. Ribosomes, together with the endoplasmic reticulum (ER), are the key drivers of protein synthesis, folding, maturation and sorting, while the proteasome plays a pivotal role in terminating the existence of thousands of proteins that are misfolded, damaged or otherwise obsolete. The synthesis, structure and function of these dedicated machines has been studied for decades, however, much less is understood about the mechanisms that control and execute their own turnover. Autophagy, an evolutionarily conserved catabolic pathway, mediates degradation of a large variety of cytosolic substrates, ranging from single proteins to entire organelles or multi-subunit macromolecular complexes. In this review, we focus on selective autophagy of three key components of the protein homeostasis machinery: ribosomes, ER and proteasomes, through the selective autophagy pathways of ribophagy, ER-phagy, and proteaphagy. We discuss newly discovered mechanisms for the selective clearance of these substrates, which are often stress-dependent and involve specialized signals for cargo recognition by a growing number of receptors. We further discuss the interplay between these pathways and their biological impact on key aspects of proteome homeostasis and cellular function in health and disease.

## Introduction

The delicate intracellular balance between the generation of newly synthesized proteins and their timely disposal, is commonly referred to as protein homeostasis. Maintaining this global equilibrium is essential in guiding and preserving normal cellular function, while the dysregulation of protein homeostasis is broadly causative of a wide range of diseases (Hetz and Saxena, 2017; Gonzalez-Teuber et al., 2019). Overall proteome quality control is regulated at multiple levels through sophisticated and dynamic mechanisms, where key machineries for protein synthesis and degradation work in parallel to maintain this critical balance. Ribosomes, both freely cytosolic as well as connected to the endoplasmic reticulum (ER), direct protein translation in a highly controlled manner in close collaboration with multiple co-factors. Besides its role in calcium and lipid homeostasis, the ER, together with its numerous resident chaperones and enzymes, surveys and facilitates the critical steps of protein maturation, folding and sorting, particularly of secretory and membrane proteins (Gomez-Navarro and Miller, 2016; Hwang and Qi, 2018). It also serves in robust protein quality control by ensuring the removal of nascent misfolded polypeptides by the proteasome, through the process of ER-associated degradation (ERAD) (Christianson and Ye, 2014). Beyond its role in ERAD, the proteasome is a highly sophisticated protease complex and a key regulator of protein destruction of a large majority of cellular proteins via the ubiquitin proteasome system (UPS). Often overlooked is the fact that these protein homeostasis machineries are themselves under homeostatic control, and have limited and highly variable half-lives. Especially under conditions of cellular stress, ribosomes, ER and proteasomes are substrates for selective degradation through complex mechanisms that are only recently beginning to emerge.

Macroautophagy (hereafter autophagy) is an evolutionarily conserved catabolic process in all eukaryotes, which mediates intracellular recycling of cytoplasmic components in order to maintain cellular homeostasis (Dikic and Elazar, 2018; Levine and Kroemer, 2019). This degradation pathway involves the sequestration of intracellular material within double-membrane vesicles called autophagosomes, which eventually fuse with vacuoles (in yeast and plants) or lysosomes (in metazoans), where the cargo is degraded by resident hydrolases (Lawrence and Zoncu, 2019). Autophagy takes place during standard physiological conditions and in response to different types of stress, where it ensures intracellular clearance of damaged or superfluous organelles and proteins. Hereby, it plays a crucial role in cellular physiology and is generally regarded as protective against a wide variety of diseases including neurodegeneration, cancer, infections, and cardiovascular disorders (Levine and Kroemer, 2019).

Autophagy was formerly considered to be a non-selective, bulk degradation pathway involving random uptake of cytoplasm by phagophores (the precursors to autophagosomes), however, in recent years, tremendous progress has been made in understanding differential cargo targeting by autophagy through a process known as selective autophagy (Johansen and Lamark, 2019). Autophagy can selectively target specific cellular components, including organelles such as the ER, mitochondria, peroxisomes or lysosomes, as well as larger protein complexes such as proteasomes, ribosomes or protein aggregates (Kirkin and Rogov, 2019). A unifying principle, common to all types of selective autophagy, is the requirement of a receptor for specific cargo recognition. Selective autophagy receptors link the cargo to the autophagy machinery, through interaction with the Atg8 family proteins (Atg8 in plants and yeast, LC3/GABARAP in mammals), which are anchored in the expanding phagophore via lipidation (Rogov et al., 2014). The receptor-Atg8 interaction is mediated by so-called Atg8-interacting motifs (AIM) or LC3-interacting region (LIR) motifs, as well as some additional newly identified interaction domains (Rogov et al., 2017; Johansen and Lamark, 2019; Marshall et al., 2019). So far, of the almost 20 different types of selective autophagy that have been described, nearly half of them are ubiquitin-driven, including the processes of mitophagy, xenophagy, and aggrephagy (Khaminets et al., 2016). In this context, receptors directly bind the ubiquitin chains present on the cargo surface, through a ubiquitin-binding domain. Yet in other types of selective autophagy, including ER-phagy, the potential involvement of ubiquitin as signaling molecule is unclear and remains a topic for further investigation (Wilkinson, 2019). An additional emerging feature of autophagy receptors, besides cargo recognition, is to control the spatiotemporal formation of autophagosomes. Several receptors, including p62 and NDP52, have been described to promote autophagosome formation at the site of their cargo through the interaction with ULK1 and FIP200 (Ravenhill et al., 2019; Turco et al., 2019; Vargas et al., 2019).

**FIGURE 1:**
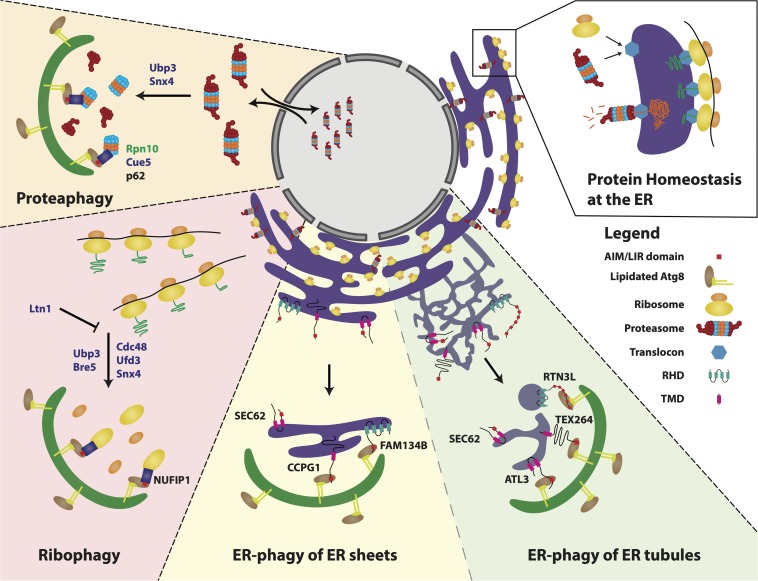
Selective autophagy of the protein homeostasis machinery. Ribosomes, proteasomes and the ER monitor and maintain protein homeostasis to ensure cellular functionality. These machineries are themselves targeted by selective autophagy as a means to regulate balanced cellular homeostasis and functionality. Proteasomes are degraded through the process of *proteaphagy* in response to starvation and/or proteasome inhibition. This process is conserved from yeast to mammals, yet with several mechanistic differences. In yeast, Ubp3 and Snx4 play key roles in triggering proteasome degradation. Identified proteaphagy receptors include Rpn10 in plants, Cue5 in yeast and p62 in mammals. *Ribophagy* of the small and large ribosomal subunits is induced by different stress conditions in yeast and mammals, including starvation/mTORC1 inhibition. In yeast, ribosome de-ubiquitination by the Ubp3 complex (comprising Ubp3, Bre5, Cdc48, and Ufd3) leads to degradation of the large subunit, which is antagonized by Ltn1-mediated ubiquitination. In humans, the ribophagy receptor NUFIP1 links ribosomes to the autophagosome to direct their degradation. FAM134B, RTN3L, SEC62, CCPG1, ATL3 and TEX264 have been identified as mammalian ER-phagy receptors. FAM134B and CCPG1 are implicated in ER maintenance of polarized cells, such as sensory axons and pancreatic acinar cells and are preferentially involved in *ER-phagy of ER sheets*. TEX264, RTN3L and ATL3 have been attributed roles in *ER-phagy of ER tubules*. TEX264 induces ER membrane engulfment from ER tubule three-way junctions by promoting autophagosome growth from these sites. RTN3L induces tubule fragmentation, leading to subsequent engulfment and degradation. SEC62 is essential for ER recovery after stress conditions with no clear preference to either ER sheets or tubules. ER sub-domain receptor preferences still require further experimental evidence, hence the division between the two is depicted by a less prominent stippled line. *Zoom in far right*: Protein homeostasis at the ER is coordinated by ribosomes and proteasomes that interact with the translocon complex to deliver newly synthesized proteins to the ER or receive proteins for degradation, respectively. Abbreviations: Ubiquitin carboxyl-terminal hydrolase 3 (Ubp3), Sorting nexin 4 (Snx4), 26S proteasome non-ATPase regulatory subunit homolog (Rpn10), Coupling of ubiquitin to ER degradation protein 5 (Cue5), mammalian target of rapamycin complex 1 (mTORC1), Ubp3-associated protein Bre5/Brefeldin A sensitivity protein 5 (Bre5), Cell division control protein 48 (Cdc48), Ubiquitin fusion degradation protein 3 (Ufd3), E3 ubiquitin-protein ligase listerin (Ltn1), Nuclear fragile X mental retardation interacting protein 1 (NUFIP1), Family with sequence similarity 134 (FAM134B), Cell cycle progression protein 1 (CCPG1), Secretory translocation protein (SEC62), Testis-expressed protein 264 (TEX264), Reticulon 3 long isoform (RTN3L), Atlastin 3 (ATL3), reticulon homology domain (RHD) and transmembrane domain (TMD). Proteins in green (plants), proteins in blue (yeast), proteins in black (humans).

In this review, we discuss the latest findings, which describe how and when autophagy can be used to selectively degrade the protein homeostasis safe-keepers: ribosomes, ER and proteasomes (Figure 1). Although these represent separately defined pathways, their physical and functional interplay is discussed, together with their implications for protein homeostasis in health and disease.

## Ribophagy

As the core of the translational machinery, the ribosome is a key complex that mediates proper decoding of the genome in space and time and thus ensures correct cellular functionality. The eukaryotic ribosome is a highly conserved complex composed of four ribosomal RNAs (rRNAs) and close to 80 ribosomal proteins. The small subunit (40S) is composed of the 18S rRNA and 33 ribosomal proteins, while the large subunit (60S) comprises three rRNAs (28S, 5.8S, and 5S) and 46 ribosomal proteins (Peña et al., 2017). Ribosome biogenesis is an energy consuming process that requires more than 200 additional factors to ensure proper rRNA folding and incorporation of ribosomal proteins into mature ribosomes (Peña et al., 2017; Klinge and Woolford, 2019).

In recent years, we have learned that the ribosome pool is heterogenous in its composition and that numerous inherent ribosome properties can promote preferential translation of distinct cellular mRNAs (Genuth and Barna, 2018; Emmott et al., 2019). Ribosome heterogeneity stems from various factors, including sequence variants and chemical modifications of the rRNA (Parks et al., 2018; Taoka et al., 2018), post-translational modifications of ribosomal proteins (Simsek and Barna, 2017; Imami et al., 2018) and stoichiometric differences in ribosomal protein composition (Shi et al., 2017). Additionally, the differential subcellular localization of ribosomes and target mRNAs contributes to the concept of localized translation, especially relevant in highly polarized cells, such as intestinal epithelium or neurons (Jung et al., 2014; Moor et al., 2017).

While increasing knowledge continues to reveal the complexity in key areas of ribosome biogenesis, structure and function, little is currently known about the turnover of ribosomes and its impact on cellular homeostasis, development and disease. The UPS has been shown to rapidly degrade excess ribosomal proteins that are not incorporated into functional ribosomes (Sung et al., 2016; Pelletier et al., 2017). This process is crucial for cell proliferation, since several unincorporated ribosomal proteins signal cell cycle arrest (Zhou et al., 2015). Yet in their assembled form, ribosomal subunits cannot be dealt with by the proteasome, and other means must be employed to degrade these large macromolecular complexes. Below, we discuss emerging evidence of selective ribosome degradation by the autophagy pathway.

### Ribophagy in Yeast

Ever since the early detection of autophagic vesicles by transmission electron microscopy in the 1950s, ribosomes have been found inside autophagosomes (Eskelinen et al., 2011). It was long-assumed that these autophagosome-engulfed ribosomes were the result of bulk cytoplasmic degradation, until 2008, where Kraft et al. (2008) introduced the concept of selective ribosome degradation by the process coined “ribophagy.” This pioneer study in yeast showed that proteins of the large and small subunits are degraded during nutrient starvation in a manner dependent on Atg8 lipidation and Atg1 activity. Using an image-based screen of yeast mutants, the Ubp3 and Bre5 de-ubiquitinase complex was shown to be specifically required for degradation of the large ribosomal subunit (Kraft et al., 2008). In follow-up studies, the Ubp3 and Bre5 binding partners, Cdc48 and Ufd3, were identified as additional players in this process, as well as γ-Glutamyl kinase (Ossareh-Nazari et al., 2010; Tatehashi et al., 2016). The Ubp3 complex de-ubiquitinates lysine 74 on Rpl25, the same residue that is ubiquitinated by the ribosome associated E3 ligase Ltn1. Interestingly, Ltn1 is also known for its role in ribosome-associated quality control (RQC) (Bengtson and Joazeiro, 2010; Ossareh-Nazari et al., 2014), a protein synthesis surveillance mechanism that, in case of ribosome stalling, initiates proteasomal degradation of the nascent polypeptide (Joazeiro, 2019). While Ltn1 depletion alone does not influence ribophagy during nutrient starvation, it restores ribosome degradation in a *Ubp3* null background. This antagonistic interplay between Ltn1 and the Ubp3 complex, through competition for the same site on Rpl25, was the first evidence of a dynamically regulated, specific ribophagy signal. The specificity of this signal was further supported by the lack of effect of Ubp3 on bulk autophagy or on the small ribosomal subunit, suggesting the existence of distinct machinery for the turnover of each subunit (Kraft et al., 2008; Ossareh-Nazari et al., 2010).

Together, these findings led to a suggested model, in which the ubiquitination of Rpl25 serves to protect ribosomes from autophagy-mediated degradation. Upon starvation, Ltn1 expression was shown to be largely decreased (Ossareh-Nazari et al., 2014), likely contributing to the stress-induced dynamics of this pathway. In contrast to other forms of selective autophagy, where cargo ubiquitination generally signals for selective engulfment by the autophagosome (Dikic and Elazar, 2018), ribophagy intriguingly seems to involve the removal of a ubiquitin mark as the trigger, at least in yeast. Still, several aspects remain unclear. For instance, it is not known how the de-ubiquitinated Rpl25 is recognized by the autophagy machinery or whether the removal of this post-translational modification may unmask an as yet unidentified signal. Moreover, the distinct mechanisms for degradation of the two subunits suggests the requirement for their dissociation prior to degradation, an area for future exploration.

### Ribophagy in Humans

Several findings over the last years have confirmed the occurrence of autophagy-mediated ribosome turnover in human cells. For instance, mass spectrometry studies of isolated autophagosomes have revealed ribosomal proteins as autophagic cargo in PANC-1, MCF-7 and HeLa cells (Mancias et al., 2014; Le Guerroué et al., 2017). A pulse/chase SILAC-based approach in MCF-7 cells under conditions of autophagy induction and/or inhibition, additionally revealed unique and specific degradation patterns from averaged data of 39 large and 27 small subunit ribosomal proteins (Gretzmeier et al., 2017). Importantly, the kinetics of ribosome degradation appeared to be different from that of other cytoplasmic proteins and mitochondria, distinguishing this process from other forms of selective or bulk autophagy (Kristensen et al., 2008).

While we have a growing mechanistic understanding of ribophagy in yeast, this process was only recently described in human cells. Making use of the pH-sensitive fluorophore Keima (Katayama et al., 2011) to monitor lysosomal localization of tagged ribosomes in HCT116 and HEK293T cells, An and Harper demonstrated that degradation of small and large subunit proteins is triggered by starvation and by the mTOR inhibitor Torin1 (An and Harper, 2017). This is blocked by inhibiting autophagy initiation through *Beclin 1* (*BECN1*) knockout and phosphatidylinositol 3-kinase VPS34 inhibition by SAR405, as well as by lysosomal inhibition using bafilomycin A1. Interestingly, while starvation and Torin1-induced degradation of RPL28 was reduced in *ATG5* knockout cells, Keima-tagged RPS3 remained unaffected, highlighting potential differences in large and small subunit degradation pathways, similar to the observations from yeast (An and Harper, 2017). The dependency on Beclin1/VPS34, but not on ATG5 for RPS3 turnover, may suggest alternative or non-canonical degradation pathways, worthy of further investigation.

The selectivity of ribophagy was further assessed by testing a panel of translation inhibitors and cellular stress agents, which unlike starvation, do not broadly induce bulk autophagy. Interestingly, specific inhibitors of translation, such as cycloheximide, did not affect ribosome degradation, possibly attributed to the fact that cycloheximide locks ribosomes onto the mRNA and prevents subunit dissociation, a step, as discussed above, which may be important for ribophagy. Alternatively, translational inhibition in itself may not provide sufficient signal for ribophagy induction. In contrast, sodium arsenite, which induces stress granule formation and reversine, an inducer of chromosome mis-segregation, both stimulate ribosome degradation more specifically than mTORC1 inhibition, as assessed through comparison of multiple cargo types (An and Harper, 2017). Unlike mTORC1-dependent ribophagy, both sodium arsenite and reversine-induced degradation of small and large subunits was similarly affected in *ATG5* knockout cells, pointing toward different modes of ribosome degradation depending on the inducing stimulus. The precise triggering signal of these ribophagy-inducing agents remains unknown.

Adding to these findings, the first selective ribophagy receptor, nuclear fragile X mental retardation-interacting protein 1 (NUFIP1) was recently described in human cell lines (Wyant et al., 2018). Upon mTORC1 inhibition, this nuclear protein re-localizes and accumulates in lysosomes. Through interaction studies, Wyant et al. (2018) showed that NUFIP1 binds the ribosome, as well as LC3B but not GABARAP, through a defined LIR domain. In addition to defects in ribosome degradation, *NUFIP1* knockout cells show reduced survival during long-term starvation (72 h), which is accompanied by reduced nucleoside and arginine levels. Although the ribosome pool only contains about 3–6% of cellular protein mass, it is highly enriched for arginine and lysine, and most importantly, it constitutes to the majority of cellular RNA (Warner, 1999; Itzhak et al., 2016; Wyant et al., 2018). Also in *C. elegans*, autophagy-dependent degradation of ribosomal RNA was suggested to play a key role in maintaining nucleotide homeostasis during animal development. In this model, the loss of the lysosomal T2 family endoribonuclease RNST-2 causes accumulation of rRNA and ribosomal proteins, leading to an embryonic lethal phenotype (Liu et al., 2018). These experiments emphasize the likely physiological importance of lysosome-mediated ribosome degradation in cellular replenishment of nucleosides/nucleotides and amino acids.

The role of NUFIP1 is likely cell type- and/or stimulus-dependent, as a recent study in human trabecular meshwork cells of the eye, found NUFIP1 to translocate from the nucleus to lysosomes upon the induction of mechanical stress, without triggering ribophagy (Shim et al., 2019). As has been observed for several other types of selective autophagy, including ER-phagy and mitophagy, multiple receptors co-exist for each autophagy subtype (Kirkin and Rogov, 2019; Wilkinson, 2019), suggesting the existence of additional ribophagy receptors, depending on the initiating signal or cell type.

In summary, we have limited knowledge of ribosome degradation in yeast and mammals. Despite a functionally important de-ubiquitination signal on the large ribosomal subunit in yeast, the picture is far from complete and a selective receptor has yet to be identified. Although one receptor was recently identified in humans, we do not know how/where it binds to the ribosome, nor do we know which post-translational modifications engulfed ribosomes may carry. Interestingly, a number of studies have elucidated a broad occurrence of post-translational modifications on ribosomal proteins in response to several types of stress, including ubiquitinations and phosphorylations. For instance in response to translational stalling (Garzia et al., 2017; Juszkiewicz et al., 2018), treatment with hydrogen peroxide (Silva et al., 2015) and induction of the unfolded protein response (UPR) (Higgins et al., 2015). Apart from the well-described phosphorylation of RPS6 downstream of mTORC1 (Biever et al., 2015), a recent study found that phosphorylation of RPL12 affects translation during mitosis (Imami et al., 2018). From a functional perspective, the majority of these and other modifications remain to be understood, including their possible roles in ribosome turnover.

## Proteaphagy

Counter-balancing protein synthesis is the UPS, which is responsible for up to 80% of protein turnover in the proliferating cell, primarily degrading short lived or misfolded proteins (Zhao et al., 2015). Working in parallel with autophagy, which can eliminate a large variety of substrates, the UPS targets only single proteins and is limited by the size of the proteasome (Kocaturk and Gozuacik, 2018; Marshall and Vierstra, 2019). Besides its essential roles in maintaining proteostasis, the proteasome broadly impacts cellular processes through the removal of e.g., signaling molecules (Bard et al., 2018; Marshall et al., 2019). The eukaryotic proteasome is composed of two subunits, the core particle (CP) and the regulatory particle (RP). The CP, also referred to as the 20S, is composed of four heptameric rings that stack up to form a barrel-like structure, forming the core of the protease complex. The RP, or the 19S, contains two subcomplexes, the lid and base, that cap one or both sides of the CP. The RP is responsible for substrate recognition and unfolding, before feeding the targeted protein to the CP for degradation (Livneh et al., 2016). The specificity of the UPS is guided by ubiquitination of proteins directed for degradation. The RP base harbors proteins that recognize substrates by their ubiquitin modifications, while the RP lid removes the ubiquitin marks from substrate proteins prior to their degradation (Albornoz et al., 2019; Marshall and Vierstra, 2019). Proteasomes are highly mobile complexes that shuttle between the cytoplasm and the nucleus depending on the cell cycle, cellular growth and stress conditions (Grice and Nathan, 2016; Livneh et al., 2016).

While the function of the proteasome is well-established, the fate of its own components and the regulation of their turnover is less well understood. The initial indication of autophagy targeting proteasomes was discovered in 1995, when proteasomes were observed within autophagic vesicles and lysosomes of rat liver cells under starvation (Cuervo et al., 1995). Yet it was not until 2015, that selective autophagy of proteasomes was confirmed, and coined “proteaphagy” (Marshall et al., 2015). Proteaphagy has since been shown to occur in plants, yeast and mammalian cells and to be a highly regulated process mediated through distinct mechanisms depending on the physiological context (Marshall et al., 2015, 2016; Waite et al., 2016; Marshall and Vierstra, 2018).

### Proteaphagy in Plants

In a pioneer study by Marshall et al., it was confirmed that in *Arabidopsis Thaliana*, proteasomes are targeted by autophagy. Briefly, by GFP-tagging the CP protein Pag1 and the RP protein Rpn5a, the extent of vacuole-dependent GFP cleavage was used as a readout for proteaphagic flux. Using this assay, it was shown that autophagy of both proteasome subunits is induced upon nitrogen starvation and is dependent on Atg8 lipidation by Atg7 and Atg10 (Marshall et al., 2015). Even in fully fed plants, both proteasomal subunits accumulate in autophagy-deficient mutant strains, while the global proteasomal activity remains unchanged compared to wild type plants, suggesting a basal level of proteaphagy that mainly targets inactive proteasomes (Marshall et al., 2015). The same study showed that proteaphagy can also be induced by proteasome inactivation. Plants deficient for proteasome assembly (*rpt2a-2*, *rpt4b-2*) or treated with a proteasome inhibitor, MG132, displayed increased levels of proteaphagy, while bulk autophagy, measured by lysosomal cleavage of GFP-Atg8, remained unchanged. Distinct from starvation-induced proteaphagy, upon their inactivation, proteasomes themselves become heavily ubiquitinated. The ensuing proteaphagy is dependent on Rpn10, an integral component of the RP, which is required for recognition of ubiquitinated substrates (Marshall et al., 2015; Bard et al., 2018). Unlike other proteasomal proteins, Rpn10 can also be found as a free cytoplasmic protein, not incorporated into the proteasome (van Nocker et al., 1996; Marshall et al., 2015). Free Rpn10 accumulates on inactivated proteasomes in a ubiquitin-dependent manner and serves as a proteaphagy receptor that simultaneously binds to ubiquitinated proteasomal subunits and to Atg8, via two distinct ubiquitin-interacting motifs (UIMs) (Marshall et al., 2015, 2019). The sequence of Rpn10 and its binding motifs are highly conserved among plants, however, the yeast and human homologs of Rpn10 (PSMD4 in humans) have neither been shown to interact with Atg8 nor to impact proteaphagy (Marshall et al., 2016; Demishtein et al., 2017).

### Proteaphagy in Yeast

To date, proteaphagy is best understood in yeast, where it is specifically induced during both proteasome inhibition and nitrogen starvation, while carbon starvation, which also induces bulk autophagy, does not stimulate proteaphagy (Marshall and Vierstra, 2018). As in *Arabidopsis*, turnover of the yeast proteasome is directed by distinct, stimulus-dependent pathways, which are dependent on the core autophagy machinery (Marshall et al., 2016; Waite et al., 2016). Additionally, yeast proteaphagy depends on sorting nexin 4 (Snx4, also known as Atg24), which dimerizes with Snx41 or Snx42 to mediate turnover of both subunits during nitrogen starvation or proteasome inhibition. As this is dependent on Snx4’s capacity to bind to phosphatidylinositol 3-phosphate-containing membranes, it may function by recruiting cargo to the autophagic membrane. Snx4 is dispensable for bulk autophagy, but was shown to be required for selective autophagy of proteasomes and in fact also ribosomes (Nemec et al., 2017). An additional co-regulator of both ribophagy and proteaphagy is Ubp3, which regulates CP but not RP degradation during nitrogen starvation, through the removal of an inhibitory ubiquitin mark (Kraft et al., 2008; Waite et al., 2016). Thus, similarly to ribophagy, these findings suggest subunit-specific mechanisms for proteaphagy induction. This is further substantiated by experiments demonstrating that trapping the RP lid in the nucleus via an inducible tether does not affect the turnover of the RP base or the CP. However, some interdependency may exist in the cytoplasm, since tethering either subunit to the plasma membrane does in fact impact degradation of the other subunit (Haruki et al., 2008; Nemec et al., 2017).

Similar to observations from *Arabidopsis*, clearance of inactive proteasomes is mechanistically distinct from starvation-induced proteaphagy. Following their inhibition, proteasomes undergo extensive ubiquitination and accumulate in cytoplasmic insoluble protein deposits (IPODs), which are a prerequisite for proteaphagy (Marshall et al., 2016). Moreover, the ubiquitin binding-factor, coupling of ubiquitin to ER degradation-5 (Cue5), has been identified as an autophagy receptor of inactive proteasomes. It binds to ubiquitinated proteasomes via its CUE domain and to Atg8 via its AIM domain to sequester aggregated, inactive proteasomes for autophagic degradation (Marshall et al., 2016, 2019). Cue5 is specifically required for proteaphagy of chemically or genetically inactivated proteasomes, but does not play a role in starvation-induced proteaphagy (Marshall et al., 2016).

### Proteaphagy in Mammals

Proteaphagy has been described in mammalian cells in response to amino acid starvation. Unlike in plants and yeast, the mammalian proteasome becomes ubiquitinated upon starvation, which is essential for its degradation by autophagy (Marshall et al., 2015; Cohen-Kaplan et al., 2016; Marshall and Vierstra, 2018). The ubiquitin-modified proteasome is recognized by the well-characterized autophagy receptor sequestosome 1 (p62/SQSTM1), implicated in several types of selective autophagy (Kirkin and Rogov, 2019). p62 can act as a selective proteaphagy receptor to mediate autophagosomal uptake of proteasomes in HeLa cells. The starvation-induced recognition of the ubiquitinated proteasome by p62 is mediated by its UBA domain, while its PB1 domain is dispensable for this process (Cohen-Kaplan et al., 2016). In contrast, the PB1 domain is responsible for p62-mediated substrate delivery to the proteasome for degradation (Seibenhener et al., 2004). These findings interestingly place p62 as a decisive factor in the regulated balance between actively supporting proteasomal function vs. targeting it for lysosomal decay.

Overall, it seems that proteaphagy occurs broadly amongst different organisms, although mechanistic details differ and lack further characterization. While the existence of this process across species suggests its physiological importance, its biological implications remain largely unknown. Especially worthy of further investigation is whether proteaphagy plays a protective role in maintaining a healthy cellular proteasome pool by selectively targeting those that are dysfunctional. Future studies will clarify these and other points and reveal the potential consequences of defective proteaphagy for human development and disease.

## ER-Phagy

The ER is a versatile organelle and apart from its afore-mentioned roles in translation, folding, sorting and ERAD, it is also important for lipid synthesis and calcium storage and release (Phillips and Voeltz, 2016). Structurally the ER consists of flat membrane sheets that are covered by ribosomes (rough ER) and branched tubules that are spread throughout the cytosol (smooth ER). Generally speaking, the rough ER is the predominant location of synthesis and translocation of luminal and secretory proteins, while the smooth tubules interact with various other organelles to influence their lipid composition or calcium levels (Shibata et al., 2006; Phillips and Voeltz, 2016). However, care must be taken to avoid oversimplifying the division between these two ER-subtypes. As a dynamic organelle, the ER as a whole must adjust to accommodate the changing demands in cellular protein homeostasis. Lysosomal degradation of ER components provides a means to adjust ER volume and ensure its functionality. The selective degradation of ER via the autophagy-lysosome pathway is termed ER-phagy and can be subdivided into macro-ER-phagy and micro-ER-phagy. While macro-ER-phagy is dependent on the core autophagy machinery and is characterized by cargo engulfment into typical double-membrane vesicles, micro-ER-phagy is mainly independent of the autophagy machinery, where cargo is instead engulfed directly by endolysosomes (Wilkinson, 2019). However, recent examples illustrate that the formation of micro-ER-phagy vesicles can, in some cases, involve core autophagy proteins (Fregno et al., 2018; Loi et al., 2019). We here focus on macro-ER-phagy (from now on termed ER-phagy), by briefly reviewing some of its key molecular players and its implications for ER homeostasis. For a more detailed overview of this pathway, we refer the reader to a number of recent comprehensive reviews on ER-phagy (Grumati et al., 2018; Loi et al., 2018; Wilkinson, 2019).

### ER-Phagy Receptors

Recently, a number of independent studies have revealed the existence of several specialized ER-phagy receptors. This includes six mammalian receptors (FAM134B, RTN3L, SEC62, CCPG1, ATL3, and TEX264) (Khaminets et al., 2015; Fumagalli et al., 2016; Grumati et al., 2017; Smith et al., 2018; An et al., 2019; Chen et al., 2019; Chino et al., 2019), as well as two yeast receptors (Atg39 and Atg40) (Mochida et al., 2015). All ER-phagy receptors are ER resident membrane proteins, which, like other known autophagy receptors, bind directly to ATG8 family members through one or several AIM/LIR motifs (Rogov et al., 2014). Below follows a brief description of the six mammalian ER-phagy receptors, their ATG8 interactions and their biological roles in cellular fitness and disease.

Family with sequence similarity 134 (FAM134B) was the first ER-phagy receptor described in mammalian cells (Khaminets et al., 2015). It contains a membrane-embedded reticulon-homology domain (RHD), that allows it to bind and reshape ER membranes and a C-terminal LIR domain, both of which are crucial for its receptor function. Interestingly, cells depleted of FAM134B show a substantial increase in ER volume and its knockout *in vivo* leads to neurodegeneration in peripheral sensory neurons with an associated inflated ER phenotype (Khaminets et al., 2015). A recent study found that misfolded procollagen in the ER is recognized by calnexin, which directly interacts with FAM143B to form ER sub-domains that are degraded through ER-phagy (Forrester et al., 2019). Thus, FAM134B is a crucial ER-phagy receptor with important implications in sensory axon maintenance and collagen production.

Reticulon domain-containing proteins (RTN1-4) reside in ER tubules, where they are able to bend and shape ER membranes (Voeltz et al., 2006). A unique member of this reticulon protein family is the long isoform of RTN3 (RTN3L), recently characterized as an ER-phagy receptor, which harbors multiple LIR domains and specifically mediates ER tubule turnover (Grumati et al., 2017). RTN3L homo-dimerization leads to ER tubule fragmentation and subsequent lysosomal degradation of these fragmented tubules. Interestingly, both fragmentation and lysosomal delivery are dependent on RTN3L’s N-terminal LIR domains. Bulk autophagic flux and ER sheet degradation remain unaffected in the RTN3 pan-isoform knockout. However, re-introduction of RTN3L alone into these knockouts is sufficient to rescue ER tubule degradation, emphasizing the specificity of this receptor and highlighting the existence of distinct ER subtype degradation pathways mediated through different receptors. In contrast to *FAM134B*, *RTN3L* deletion does not evoke any apparent phenotype in mice, nor are there any known human pathologies related to this protein.

A third ER-phagy receptor is SEC62, part of the multiprotein translocon complex which imports nascent polypeptides from translating ribosomes into the ER lumen (Linxweiler et al., 2017). This transmembrane protein is required for ER-stress recovery in a manner that is dependent on a functional LIR domain at its C-terminus (Fumagalli et al., 2016). Despite the essential involvement of LC3 and the lipidation machinery proteins in SEC62-dependent ER degradation, this process does not require proteins of the canonical autophagy initiation machinery, such as ULK1, ULK2, ATG13, and ATG14. Thus, a recent study suggests that ER-phagy after stress recovery is an atypical type of piecemeal micro-autophagy, which is dependent on LC3 and SEC62 (Loi et al., 2019). Interestingly, mass spectrometry analysis of autolysosomal content revealed a selective panel of ER proteins, whose degradation is dependent on SEC62, including ER chaperones and protein disulfide isomerases, which are excluded from autophagosomes in SEC62 LIR mutant cells (Fumagalli et al., 2016). Other protein substrates, including ERAD proteins, are degraded independently of SEC62 function. Moreover, SEC62 has been found to be upregulated in several types of cancer, where it is associated with increased metastatic and invasive potential, as well as higher ER stress tolerance (Greiner et al., 2011a, b; Wemmert et al., 2016). Whether SEC62’s role in re-establishing basic ER physiology via degradation of UPR proteins is related to its role in tumorigenesis remains an area for further investigation.

Cell cycle progression protein 1 (CCPG1) is another ER-resident transmembrane ER-phagy receptor that binds to ATG8 proteins via its LIR domain and additionally binds directly to FIP200, potentially linking it to the initiation of autophagosome formation at the site of the cargo (Smith et al., 2018). In support of this, in cultured cells, CCPG1 forms puncta, which are also positive for early phagophore markers including WIPI2 and ZFYVE1 (also DFCP1). Treatment with UPR inducers DTT, tunicamycin and thapsigargin were shown to drive CCPG1-dependent ER-phagy in a manner that was dependent on its binding to both ATG8 and FIP200. Interestingly, the *CCPG1* gene is UPR-responsive, as both its mRNA and protein levels are upregulated upon induction of ER stress. A pancreatic proteostasis defect was observed in a hypomorphic *CCPG1* mouse model, characterized by depolarization of acinar cells of the exocrine pancreas, which was accompanied by the accumulation of ER-produced secretory proteins and ER-luminal chaperones (Smith et al., 2018). Interestingly, apart from the pancreatic organ damage, the adult gastric epithelium displayed a similar loss of polarity, suggesting the importance of CCPG1 in proteostatic maintenance and proper function of polarized cells (Smith et al., 2018).

An additional, recently identified ER-phagy receptor is atlastin GTPase 3 (ATL3), part of the atlastin protein family (ATL1, ATL2, and ATL3), which span ER membranes via two transmembrane domains and mediate ER membrane fusion via GTP-driven conformational changes (Bian et al., 2011; Byrnes and Sondermann, 2011). Important for its function as an ER-phagy receptor, ATL3 interacts specifically with GABARAP proteins but not LC3 proteins, and its knockout reduces the degradation of tubular ER membrane proteins (Chen et al., 2019). *ATL3* mutations in humans cause hereditary sensory and autonomic neuropathy type I, which is associated with ER collapse, hallmarked by aberrantly tethered tubules (Kornak et al., 2014; Krols et al., 2018; O’Donnell et al., 2018). Interestingly, the disease-linked *ATL3* mutants lose their GABARAP binding potential and cannot mediate functional ER-phagy, possibly contributing to the partial ER network breakdown observed in patients (Chen et al., 2019).

Finally, testis expressed protein 264 (TEX264) was recently discovered as a single pass, transmembrane selective ER-phagy receptor in two independent proteomic-based studies (An et al., 2019; Chino et al., 2019). While Chino et al. (2019) identified TEX264 in a differential interactome study of wild type LC3B vs. mutant LC3B^K51A^, An et al. (2019) discovered it in a nutrient-stress dependent proteome screen. Both studies show that TEX264 localizes to the ER with a transmembrane domain and is degraded during starvation in an autophagy-dependent manner. Additionally, Chino et al. (2019) demonstrate that this receptor accumulates *in viv*o in a variety of organs in *Atg5* knockout mice. TEX264 accumulates at ER tubule three-way junctions that are already positive for ATG8 proteins. By using TEX264-APEX2 proximity labeling to detect proteins in close vicinity, the authors identified not only ATG8 proteins, but also key proteins of the canonical autophagy machinery, such as VPS34 complex proteins, WIPI2 and p62 (An et al., 2019). These findings led them to hypothesize that TEX264 localizes to autophagosome isolation membranes at ER tubules and helps to form the growing autophagosome in a zipper-like fashion along the ER membrane.

### ER-Phagy Receptors: Redundancy or Specialization?

The identification of six mammalian ER-phagy receptors intuitively raises the discussion of functional redundancy. One factor likely to contribute to the differential roles of ER-phagy receptors is their distinct binding patterns to different ATG8 proteins. For instance, when comparing the interactome of FAM134B and RTN3L, it was shown that FAM134B preferentially binds to LC3B and GABARAP-L2, while RTN3L predominantly binds to GABARAP-L1 (Grumati et al., 2017). Also, ATL3 specifically binds GABARAP- but not LC3 proteins (Chen et al., 2019). Several emerging studies suggest distinct functions for LC3 and GABARAP proteins in various steps of the autophagy pathway, ranging from autophagosome formation to autophagosome-lysosome fusion (Weidberg et al., 2010; Nguyen et al., 2016; Vaites et al., 2018). Similarly, some newly identified subtypes of selective autophagy depend specifically on selected ATG8 family members (Holdgaard et al., 2019). The evolution of the six human homologs from the yeast Atg8 may reflect the need to meet increased complexity in higher organisms, with critical roles in differential cargo recruitment. The extent to which these differences in ATG8 binding contribute to the distinct functions of the ER-phagy receptors remains a subject of future study.

In a functional comparison of all receptors for their individual contributions to ER-phagy during starvation, Chino et al. (2019) found that TEX264, FAM134B and CCPG1 had the strongest impact on ER-phagy, while SEC62 and RTN3L lacked clear effects in HeLa cells. The lack of effect of SEC62 and RTN3L might be attributed to their more specialized roles in recovery from ER stress in the case of SEC62 (Fumagalli et al., 2016) and degradation of fragmented tubules for RTN3L (Grumati et al., 2017). In line with this, Grumati et al. (2017) did not observe any direct interaction or functional interdependency between FAM134B and RTN3L. In HeLa cells, a triple knockout of *TEX264*, *FAM134B*, and *CCPG1* nearly mimics a *FIP200* knockout with regards to the potency of ER-phagy induction, as assessed by an ER-resident tandem RFP-GFP reporter, with the largest phenotypic contribution attributed to TEX264 (Chino et al., 2019). Although quantitative proteomics indicate an accumulation of ER resident proteins during amino acid starvation in *TEX264* knockout cells, these cells did not display an enlarged ER area upon starvation or ER stress as previously seen for CCPG1 depletion (Smith et al., 2018; An et al., 2019). Besides these functional differences, which are likely attributed to preferences of some receptors for certain ER sub-domains, a largely unresolved issue regards ER-phagy triggering “eat-me” signals. Here, the extent to which post-translational modifications of cargo and/or receptors is involved, remains a key area for future exploration. Undoubtedly, these and other studies will shed further light on the differences in ER-phagy receptors and ER-phagy subtypes, along with their individual implications in the development of disease.

## Ribophagy, Proteaphagy and ER-Phagy: Functional Interplay in Protein Homeostasis

The cell is constantly challenged to deal with a shifting balance between protein production versus protein destruction in order to maintain and shape the dynamic state of proteome equilibrium. One of the means to acquire this, is through the selective turnover of protein homeostasis safe-keepers, as described in this review and summarized in Figure 1. This occurs in a highly context-dependent manner, involving a number of co-regulatory factors and receptors, both in basal conditions, as well as in response to a broad variety of cellular stress.

Although ribophagy, proteaphagy and ER-phagy are known as distinct, separately regulated pathways, noteworthy connections exist. In fact, the ER serves as a platform for both ribosomes and proteasomes, accounting for their commonly observed intracellular co-localization. The SEC61 channel, which forms the core of the translocon complex, binds ribosomes to the ER, giving them their “rough” appearance. Yet it also binds to the proteasome via the 19S RP and accordingly, a large fraction of cytoplasmic proteasomes associates with the ER membrane (Kalies et al., 2005). Thus, ribosomes and proteasomes can physically compete for binding to the SEC61 channel in a counterbalance between nascent peptide synthesis versus degradation upon misfolding (Kalies et al., 2005; Ng et al., 2007; Kaiser and Römisch, 2015).

The physical association of ribosomes and proteasomes at the ER deserves considerable attention in future investigations of selective autophagy. It raises the issue of by-stander autophagy, in which nearby components can be captured by autophagosomes alongside selective cargo. In line with this, a substantial amount of by-stander autophagy is reported even after relatively specific ribophagy-inducing treatments, such as sodium arsenite and reversine (An and Harper, 2017). It is possible that this is due to a more general stress-response, which in addition to ribophagy, induces bulk autophagy in parallel. Yet the close association of ribosomes with multiple additional cytoplasmic components, is also likely to play a role. Moreover, since 15–35% of ribosomes are present at the ER membrane (Attardi et al., 1969; Itzhak et al., 2016), a better understanding of the distinction between ribosome degradation via ribophagy vs. their possible degradation through rough ER uptake during ER-phagy, is lacking. Yet the common observation of free, non-membrane-bound ribosomes within autophagic vesicles by electron microscopy, suggests a clear distinction between these (Eskelinen, 2008; Zhuang et al., 2013, 2017). It is possible that mechanisms for selective cargo sorting and/or exclusion may occur. For instance, a recent study identified SEC24C, essential for sorting cargo into COPII vesicles, to be important for FAM134B and RTN3L mediated ER-phagy (Cui et al., 2019). SEC24C could potentially contribute to selective cargo-sorting during ER-phagy, in a similar fashion to its COPII vesicle-related sorting. Importantly, the issue of by-stander autophagy can be largely extrapolated to several additional types of selective autophagy and emphasizes the importance of always controlling for alternative cargo degradation. It also stresses the need to further dissect the specific mechanisms of cargo selection and/or exclusion, including a better understanding of context-dependent ubiquitin usage during various types of selective autophagy.

It is noteworthy that common regulators of proteaphagy and ribophagy exist, such as Ubp3 and Snx4, with identified roles in both processes in yeast (Kraft et al., 2008; Waite et al., 2016; Nemec et al., 2017). Additionally, Ubp3 has been identified as a negative regulator of mitophagy in yeast (Müller et al., 2015). This mechanistic overlap may imply a biological cross-talk between these degradation pathways, which is a subject worthy of further investigation. Additional regulators have several independent biological roles, potentially linking functions between different pathways. For instance, the E3 ubiquitin ligase Ltn1, a starvation-responsive signaling factor for ribophagy, also acts in the process of RQC by marking nascent polypeptide chains with ubiquitin to signal their proteasomal degradation (Bengtson and Joazeiro, 2010; Ossareh-Nazari et al., 2014; Joazeiro, 2017). As RQC is often linked to ribosomal stalling and/or translation of malfunctional polypeptides, it would be intriguing to investigate the interplay of this process with ribophagy, potentially via Ltn1.

## Concluding Remarks

While significant functional consequences of ER-phagy subtypes are emerging, including the importance in sensory axon maintenance, collagen production and cellular polarization (Khaminets et al., 2015; Smith et al., 2018; Forrester et al., 2019), we lack insight toward the cellular and physiological consequences of selective proteaphagy and ribophagy in different contexts. Although proteasomes and ribosomes are relatively stable complexes with half-lives ranging from 16 h in mouse embryonic fibroblasts to over 2 weeks in rat liver cells and other cell types (Liebhaber et al., 1978; Tanaka and Ichihara, 1989; Tomita et al., 2019), their destruction is rapidly enhanced under conditions of stress. This will undoubtedly impact fundamental cellular processes that have yet to be characterized in detail. In fact, the abundance of proteasomes and ribosomes suggests an enormous potential for lysosome-mediated replenishment of amino acids and especially nucleotides through rRNA recycling. Moreover, the impact of proteaphagy and ribophagy in shaping the functional pools of proteasomes and ribosomes, respectively, is unknown. As increasing light is shed on ribosome heterogeneity and its functional implications for translation and cellular fate (Genuth and Barna, 2018; Emmott et al., 2019), it will be interesting to reveal what role ribophagy could play in altering the composition of the ribosome pool and to understand how this may impact cellular translation. Ultimately, strengthening these avenues of research will shed light on valuable therapeutic intervention opportunities in several areas, including cancer and neurodegeneration.

## Author Contributions

CB, SB, and LF conceived the review topic, discussed its contents, and wrote the manuscript.

## Conflict of Interest

The authors declare that the research was conducted in the absence of any commercial or financial relationships that could be construed as a potential conflict of interest.
